# The Efficacy of a Multidisciplinary Approach and Diagnostic–Therapeutic Algorithm for Vertebral Metastases with Spinal Cord Compression

**DOI:** 10.3390/medicina60071020

**Published:** 2024-06-21

**Authors:** Rossella Rispoli, Fabrizia Giorgiutti, Claudio Veltri, Edi Copetti, Pietro Imbruce’, Giorgia Iacopino, Barbara Cappelletto

**Affiliations:** Spine and Spinal Cord Surgery Unit, ASUFC, University Hospital Santa Maria della Misericordia of Udine, 33100 Udine, Italy; fabrizia.giorgiutti@asufc.sanita.fvg.it (F.G.); claudio.veltri@asufc.sanita.fvg.it (C.V.); pietro.imbruce@asufc.sanita.fvg.it (P.I.); barbara.cappelletto@asufc.sanita.fvg.it (B.C.)

**Keywords:** metastatic spinal disease, spinal cord compression, diagnostic–therapeutic algorithm, multidisciplinary approach, surgical intervention, radiation therapy

## Abstract

*Background and Objectives*: Metastatic spinal cord compression represents a substantial risk to patients, given its potential for spinal cord and/or nerve root compression, which can result in severe morbidity. This study aims to evaluate the effectiveness of a diagnostic–therapeutic algorithm developed at our hospital to mitigate the devastating consequences of spinal cord compression in patients with vertebral metastases. *Materials and Methods*: The algorithm, implemented in our practice in January 2022, is based on collective clinical experience and involves collaboration between emergency room physicians, oncologists, spine surgeons, neuroradiologists, radiation oncologists, and oncologists. To minimize potential confounding effects from the COVID-19 pandemic, data from the years 2019 and 2021 (pre-protocol) were collected and compared with data from the years 2022 and 2023 (post-protocol), excluding the year 2020. *Results*: From January 2022 to December 2023, 488 oncological patients were assessed, with 45 presenting with urgency due to suspected spinal cord compression. Out of these, 44 patients underwent surgical procedures, with 25 performed in emergency settings and 19 cases in elective settings. Comparatively, in 2019 and 2021, 419 oncological patients were evaluated, with 28 presenting with urgency for suspected spinal cord compression. Of these, 17 underwent surgical procedures, with 10 performed in emergency scenarios and 7 in elective scenarios. Comparing the pre-protocol period (years 2019 and 2021) to the post-protocol period (years 2022 and 2023), intrahospital consultations (commonly patients neurologically compromised) for spine metastasis decreased (105 vs. 82), while outpatient consultations increased remarkably (59 vs. 124). *Discussion*: Accurate interpretation of symptoms within the context of metastatic involvement is crucial for patients with a history of malignancy, whether presenting in the emergency room or oncology department. Even in the absence of a cancer history, careful interpretation of pain characteristics and clinical signs is crucial for diagnosing vertebral metastasis with incipient or current spinal cord compression. Early surgical or radiation intervention is emphasized as it provides the best chance to prevent deficits or improve neurological status. Preliminary findings suggest a notable increase in both the number of patients diagnosed with suspected spinal cord compression and the proportion undergoing surgical intervention following the implementation of the multidisciplinary protocol. The reduced number of intrahospital consultations (commonly patients neurologically compromised) and the increased number of visits of outpatients with vertebral metastases indicate a heightened awareness of the issue, leading to earlier identification and intervention before neurological worsening necessitating hospitalization. *Conclusions*: A comprehensive treatment planning approach is essential, and our multidisciplinary algorithm is a valuable tool for optimizing patient outcomes. The protocol shows potential in improving timely management of spinal cord compression in oncological patients. Further analysis of the factors driving these changes is warranted. Limitations: This study has limitations, including potential biases from the retrospective nature of data collection and the exclusion of 2020 data due to COVID-19 impact. To enhance the robustness of our results, long-term studies are required. Moreover, the single-center study design may limit the validity of the findings. Further multicenter studies would be beneficial for validating our results and exploring underlying factors in detail.

## 1. Introduction

The prevalence of secondary tumor localizations involving the spinal column is notable, accounting for approximately 60% of cases [1]. This phenomenon is attributed to the rich vascular supply and extensive lymphatic drainage of vertebral bones.

Advances in chemotherapy and radiation therapy have contributed to improved survival rates among oncological patients, resulting in a rise in the incidence of vertebral metastases [2,3]. Currently, spinal metastases are identified in approximately 20% of all oncological patients [4].

Cancers originating from the lung, breast, and prostate exhibit a higher propensity to metastasize to the spine, accounting for over 60% of cases; in about 7% of instances, the primary tumor remains unidentified [5].

Metastatic disease affecting the spine can result in bone fractures, instability, and metastatic spinal cord compression (MSCC). The latter is a particularly devastating complication, occurring in 2.5–10.0% of cancer patients and 40% of individuals with bone metastases. While metastatic spinal cord compression is more common in patients with a known primary cancer diagnosis, it can also serve as the initial presentation of malignancy in cases where cancer has not yet been diagnosed [6].

The prognosis for MSCC is better if it is treated before the onset of paralysis. However, alarmingly, up to 50% of patients lose the ability to walk before receiving a diagnosis [7].

Timely identification and treatment of metastatic spinal cord compression are imperative in preventing irreversible neurological injury, alleviating pain, and preserving patients’ mobility, function, and independence. Therefore, early detection and intervention are crucial for optimizing outcomes and maintaining patients’ quality of life in the face of metastatic spinal cord compression [8].

Effective management of spinal metastases requires a multidisciplinary approach, harnessing the expertise of various specialists for prompt diagnosis of patients with spinal metastases and cord compression, followed by comprehensive support post-diagnosis. We developed an algorithm aimed at reducing and preventing irreversible neurological deficits and the devastating consequences of spinal cord compression through timely and coordinated intervention.

## 2. Materials and Methods

**Figure 1:**
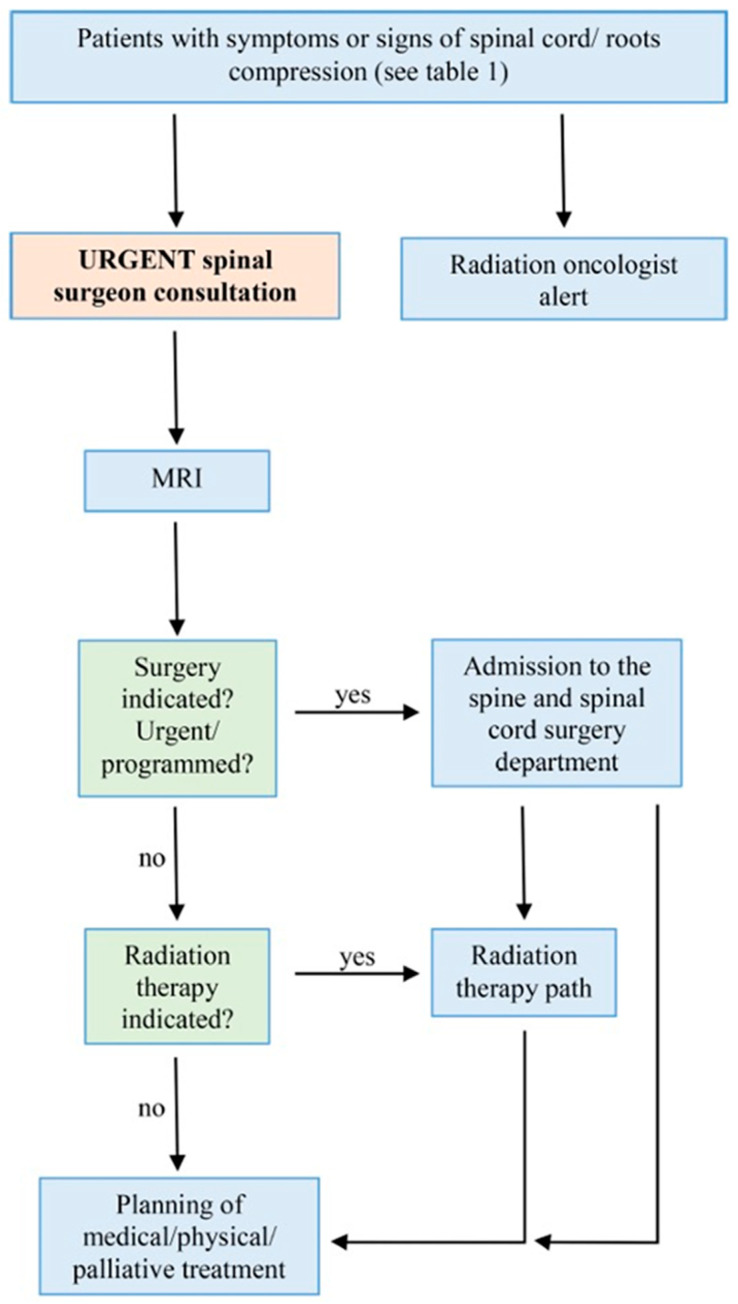
Diagnostic–therapeutic algorithm for patients with metastatic roots or spinal cord compression (MSCC).

The protocol for managing cases of metastatic spinal cord compression (MSCC) is a comprehensive, multidisciplinary approach that involves collaboration among several key specialists: the emergency room physician, spine surgeon, neuroradiologist, radiation oncologist, and oncologist. The management algorithm, illustrated in Figure 1, guides the process by outlining specific steps based on the recognition of symptoms associated with spinal cord compression.

### 2.1. Initial Recognition and Alert Symptoms

The responsibility of initial symptom recognition falls on the emergency room physician or the oncologist. Prompt identification is crucial for triggering the subsequent steps of the flowchart.

Key alert symptoms that should raise suspicion for MSCC include the following:

- Nocturnal neck or back pain: persistent pain that worsens at night;
- Axial mechanical pain: pain in the spine that is exacerbated by movement and alleviated by rest;
- Sudden onset of axial pain: acute, severe pain in the spine;
- Radicular pain: pain radiating to the arms or legs, which may be accompanied by numbness, tingling, or dysesthesia;
- Walking or balance difficulties: new or worsening problems with walking or maintaining balance;
- Weakness in arms/hands: muscle weakness affecting one or more muscles in the arms or hands.
- Bladder or bowel control disorders: issues such as urinary retention or incontinence.

When at least one of these clinical manifestations is observed, the emergency doctor notifies the spine surgeon and the radiation oncologist to evaluate the patient and trigger additional assessments and management.

### 2.2. Role of the Spine Surgeon and the Radiation Oncologist

Upon notification, the spine surgeon assumes responsibility for the patient’s care. The initial steps involve the following:

- Assessing neurological deficits: evaluating the patient for any motor or sensory deficits to determine the extent of spinal cord/root compression;
- Collaboration with the neuroradiologist: working closely with the neuroradiologist to arrange for timely magnetic resonance imaging (MRI).

Based on the findings from the MRI, the spine surgeon and the radiation oncologist collaborate to develop a tailored treatment strategy that addresses the specific needs of the patient. This strategy may include a combination of surgical intervention, targeted radiation therapy, and other supportive treatments to optimize the patient’s outcomes and ensure the most effective management of their condition.

Surgical intervention aims to decompress the spinal cord and stabilize the spine. Surgery is recommended in case of mechanical instability, significant neurological impairment, or when the tumor is scarcely responsive to radiation. The “separation surgery”, a surgical method to separate the anterior sulcus in the spinal canal from the posterior edge of the vertebral body is sometimes necessary to build the radiotherapy gradient zone. This technique allows high doses to be delivered to the target in a highly conformal fashion, suitable for stereotactic radiosurgery stereotactic radiosurgery (SRS) and stereotactic body radiotherapy (SBRT).

Radiation therapy is advised for radiosensitive tumors to reduce tumor size and alleviate spinal cord compression and, finally, to reduce pain.

Some cases may require both surgical and radiation treatments depending on the severity and specific characteristics of the spinal cord compression.

### 2.3. Role of the Oncologist, the Physiatrist, and the Palliative Care Physician

In the algorithm, the oncologist plays a critical role in managing systemic aspects of the patient’s cancer. This involves administering systemic therapy such as chemotherapy, hormonal therapy, or targeted therapy. These treatments are designed to address both the primary malignancy and any metastatic sites, ensuring comprehensive management of the disease.

Following initial treatment, rehabilitation and supportive care are vital in maximizing recovery and maintaining quality of life. Supportive care encompasses pain management, nutritional support, psychological counseling, and social services to address the holistic needs of the patient and provide emotional and practical support throughout the recovery process.

To minimize potential confounding effects from the COVID-19 pandemic, data from the years 2019 and 2021 (pre-protocol) were collected and compared with data from the years 2022 and 2023 (post-protocol), excluding the year 2020.

## 3. Results

### 3.1. Increase in Total Oncological Patients Evaluated

After the protocol was implemented, there was an increase in the total number of oncological patients evaluated. In the years 2022 and 2023, we assessed 488 patients, whereas in the years 2019 and 2021, we assessed 419 patients with metastatic spine disease.

### 3.2. Increase in Patients with Suspected Spinal Cord Compression

There was an increase in the number of patients presenting with suspected spinal cord compression after the protocol was implemented. In the post-protocol period, 45 patients with suspected spinal cord compression were evaluated, compared to 28 patients evaluated in the pre-protocol period.

### 3.3. Increase in Surgical Procedures

The number of patients undergoing surgical procedures for spinal cord compression, both in elective, delayed-urgency settings and in emergency settings, increased after the protocol was implemented. In the post-protocol period, 44 patients with spinal cord compression underwent surgery: 25 in emergency settings and 19 in elective, delayed-urgency settings. In contrast, in the pre-protocol period, we operated on 17 patients: 10 in emergency settings and 7 in elective, delayed-urgency settings.

### 3.4. Changes in Number of Intrahospital Consultations and Number of Outpatients with Spine Metastasis

There was a decrease in the number of intrahospital consultations, with 105 occurring in the pre-protocol period and 82 in the post-protocol period. Conversely, there was an increase in the number of outpatient consultations, with 59 in the pre-protocol period and 124 in the post-protocol period.

The results are summarized in Table 1.

## 4. Discussion

The treatment of spinal metastases necessitates a comprehensive and multidisciplinary approach, with careful consideration of various clinical factors and the application of specialized algorithms and scoring systems to tailor treatment plans to individual patient needs effectively [9].

The Spine Oncology Consortium (SOC) offers a framework for categorizing treatment modalities, which typically include radiotherapy, surgery, and neurointerventional procedures. These modalities can be utilized alone or in combination, depending on the individual patient’s needs and the specific characteristics of their spinal metastases [10]. Additionally, algorithms such as NOMS (neurological, oncological, mechanical, systemic) and LMNOP provide structured approaches for clinicians to evaluate and determine the most appropriate therapeutic strategies based on various clinical parameters and considerations [11,12]. Furthermore, the Spinal Instability Neoplastic Score (SINS) serves as a valuable tool for assessing the degree of instability associated with spinal metastases. This scoring system helps clinicians quantify the severity of spinal instability, guiding decisions regarding the urgency and type of intervention required to address the instability effectively while minimizing potential risks for the patient [13,14].

Guidelines recommend that clinicians remain vigilant for early signs of metastatic spinal cord compression (MSCC) in patients with cancer. Early diagnosis is crucial for prompt intervention and improved outcomes. Whole-spine magnetic resonance imaging (MRI) is advised as the preferred diagnostic modality for detecting spinal metastases and assessing the extent of spinal cord compression [15,16,17].

To facilitate early diagnosis and treatment, specific systems have been proposed to reduce delays in care. Allan et al. proposed a system that involves telephone interviews with cancer patients to identify early symptoms of MSCC. This approach aims to determine the optimal timing for MRI examinations, thereby facilitating timely intervention and improving patient outcomes [18]. Savage et al. advocate for formalized systems that provide rapid access to MRI scans through collaboration among specialists [19]. This collaborative approach aligns with guidance from the National Institute for Health and Care Excellence (NICE) and is associated with enhanced overall outcomes for patients with MSCC [20].

Our protocol is built upon three fundamental principles: spinal cord compression, timeliness, and multidisciplinarity, embodying a contemporary approach to patient care in the context of vertebral metastases. Recognizing the critical nature of spinal cord compression, our approach ensures early identification and immediate intervention to prevent irreversible neurological damage. Timeliness is crucial, with streamlined protocols facilitating rapid response from symptom recognition to treatment initiation, thereby preventing the progression of neurological deficits. Multidisciplinarity fosters collaborative care, with specialists including spine surgeons, neuroradiologists, radiation oncologists, oncologists, physiatrists, and palliative care physicians working in concert to develop individualized treatment strategies. This paradigm of holistic and patient-centered care shifts the focus from mere palliative care to active management, aiming to maximize functional recovery, minimize pain, and improve overall quality of life. By prioritizing patient needs and involving them in decision-making, healthcare providers can optimize clinical outcomes and elevate the overall patient experience for those living with vertebral metastases.

In our algorithm, the spine surgeon collaborates with the neuroradiologist to determine the optimal timing for MRI. The algorithm underscores the importance of early whole-spine MRI and advocates for a swift, integrated approach for patients with spinal cord compression. Regarding treatment, the algorithm prioritizes early surgical intervention or radiation therapy. Surgery is often the treatment of choice for patients meeting specific criteria, such as an unstable spine, single-level, or oligometastatic disease (limited number of metastases); good performance status; and the necessity for tissue sampling for disease characterization [21,22,23]. Radiotherapy is favored in patients with comorbidities, widespread disease, and a limited prognosis due to disease burden. A recently emerging and effective treatment for MSCC is radiosurgery, which delivers a very high dose of radiation to a precise target under imaging guidance. This technique enables highly focused radiation delivery, minimizing damage to surrounding healthy tissue [24,25].

The presence or absence of neurological deficits prior to treatment significantly impacts the outcome in MSCC. Treatment delays can result in irreversible neurological damage, affecting quality of life and increasing burden on healthcare resources. Patients who have experienced neurological function loss for over 24 h are unlikely to exhibit improvement and may not typically undergo surgery unless spinal stabilization is necessary for pain relief [26].

Our study findings suggest several positive outcomes following protocol implementation. The changes in care practice potentially led to a reduction of the number of patients with irreversible neurologic deficits.

The increase total number of oncological patients evaluated after the protocol compared to the pre-protocol period (488 vs. 419) indicates potentially improved access to care and increased patient referrals.

We observed an improved recognition of suspected MSCC, as evidenced by the rise in the number of patients presenting with suspected MSCC (45 vs. 28), indicating enhanced detection of the issue. This suggests a positive impact on early identification and timely intervention for such cases.

The increase in surgical procedures (44 vs. 17) reflects more appropriate management of these cases.

Emergency surgical procedures increased (25 vs. 10), indicating faster access to surgery for patients requiring urgent treatment. Data on surgery suggest that the algorithm reduced delays in care and potentially improved outcomes, highlighting the effectiveness of the protocols in expediting MSCC patient management.

The decrease in intrahospital consultations (105 vs. 82) and the increase in outpatient consultations (59 vs. 124) in the years after protocol implementation suggest potential improvements in coordination, centralization, and accessibility of oncology care. This shift towards outpatient-based care may enhance efficiency and accommodate increased patient volume, ultimately leading to better patient experiences and outcomes.

Some studies have shown a trend towards a peak in MSCC referrals on Fridays. Early referrals and expedited treatment decisions could potentially mitigate this trend. Hospitals with 7-day acute oncology services, radiology reporting, and a single point of referral, such as an MSCC coordinator, have demonstrated quicker treatment turnaround times and more consistent referrals throughout the week [27,28]. The National Institute for Health and Care Excellence (NICE) recommends that every secondary or tertiary care center should have an identified lead healthcare professional for MSCC, responsible for implementing the care pathway and coordinating diagnosis and management [20]. The activation of the protocol with the aim of standardizing procedures at any time of the day and seven days a week, as we do, is a crucial step towards ensuring timely and consistent management of MSCC.

Designing the algorithm to meet the criteria of being simple and immediate, reproducible, and practical, as well as adaptable and applicable in all cases, is essential for its effectiveness and widespread implementation. The algorithm’s simplicity and immediacy ensure that it can be quickly understood and executed by healthcare providers, facilitating timely and appropriate interventions. Its reproducibility and practicality mean that it can be consistently applied in diverse clinical settings, ensuring uniformity in patient care. Adaptability ensures that the algorithm can be tailored to the specific needs and resources of different healthcare environments, making it relevant in all cases of metastatic spinal cord compression (MSCC).

Our next project aims to expand this protocol to spoke hospitals and involve general practitioners. This expansion is crucial for significantly improving accessibility to specialized care for patients with MSCC. By integrating the protocol into peripheral hospitals and primary care settings, patients can receive timely interventions closer to their homes, reducing the reliance on referrals to centralized facilities and minimizing treatment delays. This approach streamlines care pathways, making it easier for patients to access the specialized services they need without unnecessary travel or waiting times.

Promoting early intervention through this expanded protocol is expected to enhance survival rates and functional outcomes for patients with MSCC. By addressing spinal cord compression promptly, we can prevent irreversible neurological damage, maintain mobility, and improve the overall quality of life for these patients. Additionally, this proactive approach benefits the broader healthcare system by optimizing resource utilization and reducing the burden on centralized healthcare facilities. Early intervention and streamlined care pathways not only lead to better patient outcomes but also contribute to a more efficient and sustainable healthcare delivery model.

## 5. Limitations

It is crucial to acknowledge the study’s limitations for a comprehensive understanding of its implications. Firstly, the retrospective nature of data collection introduces potential biases. Relying on previously recorded information can inadvertently lead to inaccuracies and gaps, impacting the reliability of the findings. Secondly, the exclusion of 2020 data due to the disruptive impact of COVID-19 could result in overlooking significant changes or trends from that period, potentially skewing the study’s conclusions. Lastly, the single-center design restricts the generalizability of the results, as they may not be applicable to other settings or populations, thus limiting the study’s external validity.

To bolster the robustness of the findings, there is a clear need for long-term studies. By extending the research duration, it becomes possible to capture more comprehensive trends and variations over time, thus providing a deeper understanding of the phenomenon under investigation. Additionally, conducting multicenter studies would be beneficial. These studies involve multiple institutions or locations, which not only help validate the findings across diverse populations but also allow for the exploration of underlying factors in greater detail. By addressing these limitations and adopting a more comprehensive approach, the study’s robustness and applicability can be significantly enhanced, ultimately contributing to more reliable and actionable insights for clinical practice and policymaking.

## 6. Future Directions

The application of Artificial Intelligence (AI) in medical diagnosis indeed holds immense promise for future advancements in healthcare. One of the primary advantages of AI lies in its capacity to handle vast amounts of data efficiently. Medical data, ranging from patient records to imaging studies, can be processed and analyzed at a scale and speed far beyond human capability. This ability not only expedites the diagnostic process but also enhances its accuracy by minimizing the risk of human error and biases [29].

Moreover, AI-powered diagnostic systems have the potential to revolutionize disease prediction and prevention. By leveraging predictive analytics, AI algorithms can sort through extensive patient data, including medical history, lifestyle factors, and genetic predispositions, to identify individuals at high risk for certain diseases. Early detection of these risks enables proactive intervention strategies, such as lifestyle modifications or targeted screenings, which can significantly improve patient outcomes and reduce healthcare costs.

Furthermore, the integration of AI into clinical algorithms can optimize the selection of appropriate management strategies for patients. By analyzing patient data in real-time and considering a multitude of factors, including disease severity, comorbidities, and treatment responses, AI algorithms can provide personalized recommendations tailored to each patient’s unique needs. This not only enhances the efficacy of treatment but also improves resource allocation and healthcare delivery efficiency.

Overall, the integration of AI into medical diagnosis and management represents a paradigm shift in healthcare delivery. By rationalizing diagnostic processes, improving predictive capabilities, and facilitating personalized treatment strategies, AI empowers clinicians to deliver higher-quality care while simultaneously reducing the burden on healthcare systems. As research and development in AI continue to advance, its potential to revolutionize healthcare remains boundless.

## 7. Conclusions

A comprehensive approach to treatment planning in healthcare is paramount for ensuring optimal patient outcomes, especially in complex conditions like metastatic spinal cord compression (MSCC). The proposed multidisciplinary algorithm represents a significant step forward in this endeavor, serving as a valuable tool to streamline patient management and improve overall care quality.

One of the key benefits of the multidisciplinary algorithm is its potential to enhance the detection rates of patients with MSCC. By involving specialists from various fields such as neurosurgery, oncology, and radiology, the algorithm facilitates a more thorough evaluation of patients presenting with spinal cord compression symptoms. This multidisciplinary approach increases the likelihood of identifying MSCC cases early, enabling prompt intervention and potentially preventing irreversible neurological damage.

Moreover, the algorithm promotes more timely surgical interventions for patients with MSCC. Timeliness is critical in the management of spinal cord compression as delays in treatment can lead to permanent neurological deficits. By making the decision-making process more efficient and facilitating collaboration among healthcare professionals, the algorithm ensures that patients receive surgical interventions promptly, maximizing the chances of successful outcomes and preserving neurological function.

Additionally, the multidisciplinary approach advocated by the algorithm has the potential to enhance the delivery of oncology care for patients with vertebral metastases. The algorithm facilitates a comprehensive assessment of patients’ oncological needs and coordinated treatment planning by bringing together the radiation oncologist, oncologist, physiatrist, and palliative care physician, alongside the spine surgeon and supportive care services. This holistic approach not only improves patient outcomes but also enhances the patient experience by providing seamless and integrated care.

The observed increase in the number of outpatient visits for patients with vertebral metastases suggests a growing awareness of the importance of early detection and management of spinal metastases. Further analysis of the underlying factors driving these changes would provide deeper insights into the evolving landscape of patient care. Understanding the reasons behind the increased awareness and utilization of healthcare services can inform future strategies for improving patient education, raising awareness among healthcare professionals, and optimizing resource allocation to meet the growing demand for specialized care in this patient population.

In conclusion, the multidisciplinary algorithm represents a significant advancement in the management of MSCC and vertebral metastases, offering a comprehensive approach to treatment planning that optimizes patient outcomes. By facilitating early detection, timely interventions, and coordinated oncology care, the algorithm holds the potential to transform the way these conditions are managed, ultimately improving the quality of life for patients affected by spinal metastases.

## Figures and Tables

**Table 1:** Summary of the results.

	Pre-Protocol Period (Years 2019 and 2021)	Post-Protocol Period (Years 2022 and 2023)
Oncological patients evaluated	419	488
Patients with suspected MSCC	28	45
Patients that underwent surgical procedures	17	44
Emergency/elective delayed urgency surgery	10/7	25/19
Intrahospital consultations	105	82
Outpatient consultation	59	124

## Data Availability

Data presented are original and not inappropriately selected, manipulated, enhanced, or fabricated.
